# “I Find that the Music Really Do Help Them Calm Down”: Qualitative Interviews to Support the Development of a Music‐based Application to Improve Sleep Disturbances in Persons Living with Dementia

**DOI:** 10.1002/alz.093275

**Published:** 2025-01-09

**Authors:** Darina Petrovsky, Justine S. Sefcik, Abeer Mobarki, Sharvari Kolte, Abigail Amponsah, Nancy A Hodgson, Bei Wu

**Affiliations:** ^1^ Duke University, Durham, NC USA; ^2^ Drexel University College of Nursing and Health Professions, Philadelphia, PA USA; ^3^ Rutgers University, Newark, NJ USA; ^4^ Rutgers University, New Brunswick, NJ USA; ^5^ University of Pennsylvania, Philadelphia, PA USA; ^6^ NYU Aging Incubator, New York, NY USA; ^7^ New York University, New York, NY USA

## Abstract

**Background:**

Most persons living with dementia (PLWD) experience sleep disturbances at some point during their disease. Music interventions are promising to address sleep disturbances because long‐term memory for music remains relatively preserved in PLWD. The purpose of this study was to identify the prototype features of the mobile application, entitled, “Calming Music Personalized for Sleep Enhancement in PeRsons living with Dementia” (CoMPoSER) for use among PLWD and their caregivers by conducting the first round of qualitative interviews to co‐design the content, features, and layout of the application prototype.

**Method:**

In the first step of the study (out of two), we conducted 13 virtual interviews with 20 stakeholders (seven PLWD and thirteen caregivers). Five interviews included dyads (n = 3) or triads (n = 2) of PLWD and their caregivers. We used the mHealth Usability framework for mobile applications (Tajudeen et al. 2022) to guide our directed content analysis. Based on this analysis approach, we present the findings under the framework’s constructs.

**Result:**

The average participant age was 66.7 years old (SD: 17.2). Most were women (n = 14). Twelve identified themselves as White/Caucasian, six as Black/African American, and two as Asian. The participants shared with us that it was important to design a mobile application that is simplistic and easy to navigate. For example, too many song options may confuse PLWD. Participants also talked about what their preferences for soothing colors. We captured preferred styles of music that PLWD and caregivers enjoy listening to and the high potential usefulness of the mobile application. Moreover, individuals conveyed their varying levels of comfort with technology. Some expressed high comfort levels, while others outlined the specific assistance and help information needed to feel at ease using the application.

**Conclusion:**

We used information gathered in the first step of the study to inform the second‐round interview questions. PLWD and caregivers in this study were committed to the process of developing a music‐based application to improve sleep. Their insights on simplistic features, navigation, and colors of the potential application will inform the next round of interviews, after which the prototype of the mobile application will be developed.

